# Gas Diffusion Electrodes on the Electrosynthesis of Controllable Iron Oxide Nanoparticles

**DOI:** 10.1038/s41598-019-51185-x

**Published:** 2019-10-25

**Authors:** Rafael A. Prato, Vincent Van Vught, Sam Eggermont, Guillermo Pozo, Pilar Marin, Jan Fransaer, Xochitl Dominguez-Benetton

**Affiliations:** 1 Sustainable Chemistry, VITO, Flemish Institute for Technological Research, Boeretang 200, 2400 Mol, Belgium; 2 Department of Materials Engineering, Surface and Interface Engineered Materials, Katholieke Universiteit Leuven, Kasteelpark Arenberg 44 - box 2450, 3001 Leuven, Belgium; 3 Strategic Initiative Materials in Flanders, SIM vzw, Technologiepark 935, BE-9052 Zwijnaarde, Belgium; 4 Instituto de Magnetismo Aplicado, UCM-ADIF-CSIC, Universidad Complutense de Madrid A6 22,500 Km, 28230 Las Rozas, Spain

**Keywords:** Electrochemistry, Magnetic properties and materials, Magnetic properties and materials, Design, synthesis and processing, Nanoparticle synthesis

## Abstract

The electrosynthesis of iron oxide nanoparticles offers a green route, with significant energy and environmental advantages. Yet, this is mostly restricted by the oxygen solubility in the electrolyte. Gas-diffusion electrodes (GDEs) can be used to overcome that limitation, but so far they not been explored for nanoparticle synthesis. Here, we develop a fast, environmentally-friendly, room temperature electrosynthesis route for iron oxide nanocrystals, which we term gas-diffusion electrocrystallization (GDEx). A GDE is used to generate oxidants and hydroxide *in-situ*, enabling the oxidative synthesis of a single iron salt (e.g., FeCl_2_) into nanoparticles. Oxygen is reduced to reactive oxygen species, triggering the controlled oxidation of Fe^2+^ to Fe^3+^, forming Fe_3−x_O_4−x_ (0 ≤ x ≤ 1). The stoichiometry and lattice parameter of the resulting oxides can be controlled and predictively modelled, resulting in highly-defective, strain-heavy nanoparticles. The size of the nanocrystals can be tuned from 5 nm to 20 nm, with a large saturation magnetization range (23 to 73 A m^2^ kg^−1^), as well as minimal coercivity (~1 kA m^−1^). Using only air, NaCl, and FeCl_2_, a biocompatible approach is achieved, besides a remarkable level of control over key parameters, with a view on minimizing the addition of chemicals for enhanced production and applications.

## Introduction

As nanomaterials make their way to the forefront of a variety of applications, the synthesis, characterization, and functionality of magnetic iron oxide nanoparticles (IONPs) have been the focus of significant research^1–3^. With good chemical stability^4^, outstanding redox properties, and biocompatibility^5,6^, IONPs are one of the benchmark nanomaterials for a diverse range of practical applications. In biomedical, environmental, ferrofluid, energy conversion and storage applications, diagnostics, bio-sensing, and data storage, they show encouraging potential, among other emerging uses^3,7^. Certain physicochemical characteristics of IONPs determine their properties and potential functionality. Size, dispersity, composition, and structure are amongst the aspects that must be tailored for each specific use^3,7^, yet not always accomplished.

Among the commonly found iron oxides, magnetite (Fe_3_O_4_) and maghemite (γ-Fe_2_O_3_) nanoparticles are of chief interest. They are ubiquitous in the environment and have broad bio-geochemical implications. Magnetite is the most magnetic, naturally occurring, mineral on our planet^8^. It is the iron oxide that exhibits the largest saturation magnetization (92 A m^2^ kg^−1^) and a high Curie temperature (840 K) in the bulk^4^. As nanoparticles (NPs), particle size affects their magnetic susceptibility. Below a magnetic domain size of approximately 25–30 nm, magnetite may transition from ferrimagnetic to superparamagnetic^9^. Like magnetite, maghemite has a high Curie temperature (928 K), yet displays a lower saturation magnetization (*M*_*s*_) at room temperature (up to 81 A m^2^ kg^−1^). The ultrafine particles of maghemite, like magnetite, show superparamagnetism. It has been shown that for nearly spherical shapes, the *M*_*s*_ for nanoparticles can be equal to the bulk value with negligible coercivity, implying near perfect crystals^8^. IONPs can also exhibit close to bulk magnetization at relatively large crystallite sizes of around 20 nm. Further below the ferrimagnetic-superparamagnetic transition size, the saturation magnetization decreases^3^.

IONPs, as opposed to larger ferrimagnetic particles, are better suited for preparing stable dispersions^10^, a key feature for various applications. Of crucial importance in the optimization of IONP synthesis are: control over the phase composition, synthesis of particles with ~20 nm magnetic domains, and a narrow size distribution. This size range results in an optimal magnetization, while fine control over the phase composition (Fe_3_O_4_ vs. γ-Fe_2_O_3_) may enable the formulation of precise structure-property relations^10^.

Synthesis techniques are numerous and vary in nature and focus. Sol-gel, co-precipitation, microemulsion, hydrothermal^10^, electrochemical^11^, thermolysis of precursors^12^, and spray pyrolysis, are the most extended. All of these techniques lead to different degrees of control over the aforementioned and additional properties. For many applications, the chemical co-precipitation of mixed iron salts is the most commonly found synthesis route^3^. Here we present a new synthesis method: an electrochemical process using a gas diffusion electrode, namely Gas Diffusion Electrocrystallization (GDEx).

The electrochemical formation of iron oxide nanoparticles—which typically takes place under anodic oxidation conditions—is limited by the solubility of oxygen in the electrolyte^13^. Gas diffusion electrodes (GDEs) have recently gained vast momentum, as they have proven to push current densities and selectivity, as they improve the electrochemical transformations of gases (e.g., O_2_, CO_2_, etc.) by overcoming the gas solubility limit in aqueous electrolytes^14^ and greatly reducing the gas mass-transfer constraints^15^. Although the use of GDEs is widely extended in classical fuel cells, chemical and electricity cogeneration fuel cells, microbial fuel cells, and recently in CO_2_ electrocatalysis, they have not been investigated for the formation of nanoparticles, which is the key innovation driver in this study.

In GDEx, metal ions are supplied to a triple phase boundary (liquid electrolyte, solid electrode, and a gas), wherein the electrochemical reduction of the gas (exemplified here by O_2_) takes place—provided a suitable electrode potential. This results in locally-tuning the pH of the electrolyte (via the interfacial generation of e.g., OH^–^), with the simultaneous replenishment of an *in-situ* generated highly-oxidizing agent (i.e., H_2_O_2_ and other reactive oxygen species, ROS). These conditions, collectively facilitate a reaction front, the onset for supersaturation, and hence for the reactive precipitation of the metal ions supplied (into e.g., oxides, hydroxides). The combination of the selected metal ion precursors, their concentration, and selected flowing conditions, provide an optimal environment for the formation of free nanoparticles with highly-controlled physicochemical features and properties. Iron ions were selected as the precursor to present the first proof of concept of GDEx; yet, it is valid for many more metal precursors.

Furthermore, as opposed to classical co-precipitation methods where stoichiometric amounts of Fe^3+^ and Fe^2+^ are added^16^, one of the features of GDEx, for the formation of IONPs, is the use of a single iron salt, FeCl_2_, and no other chemicals. Iron (II) ions are oxidized at the cathodic electrochemical interface by the ROS formed by the partial reduction of oxygen in the gas diffusion electrode. This electrochemically-driven processes allow for the formation of a range of iron species from the same iron salt. Fe(OH)_2_, Fe_3_O_4_, γ-Fe_2_O_3_, and FeOOH can be obtained. These structures can be precisely tailored by changing the GDEx synthesis parameters, as needed for target applications. In this manner, fine control over the phase composition is exploited, and optimal particle properties are achieved, abating a challenge in the current synthesis of IONPs. Dispersion of the nanoparticles is explored as well, from the primary size of crystallites, to particles and aggregates; size measurement techniques are contrasted to obtain a fuller fingerprint of the material. Primary sizes in the range of 5–20 nm, hydrodynamic sizes of 140 nm for the superparamagnetic particles, variable Fe_3_O_4_/γ-Fe_2_O_3_ compositions within particles, minimal coercivities, and magnetic susceptibilities of up to 73 A m^2^ kg^−1^ are shown, proving GDEx as a new nanoparticle synthesis method which can achieve a remarkable control over various physicochemical properties of the nanoparticles and the elucidation of rational structure-property relationships. In addition, GDEx represents a suitable alternative for immediately scaling-up production by enlarging the size and number of electrodes, varying the current density, assembling a continuous and flow-cell approach, or a combination of these.

## Experimental

### Synthesis procedure

#### GDEx setup

**Figure 1:**
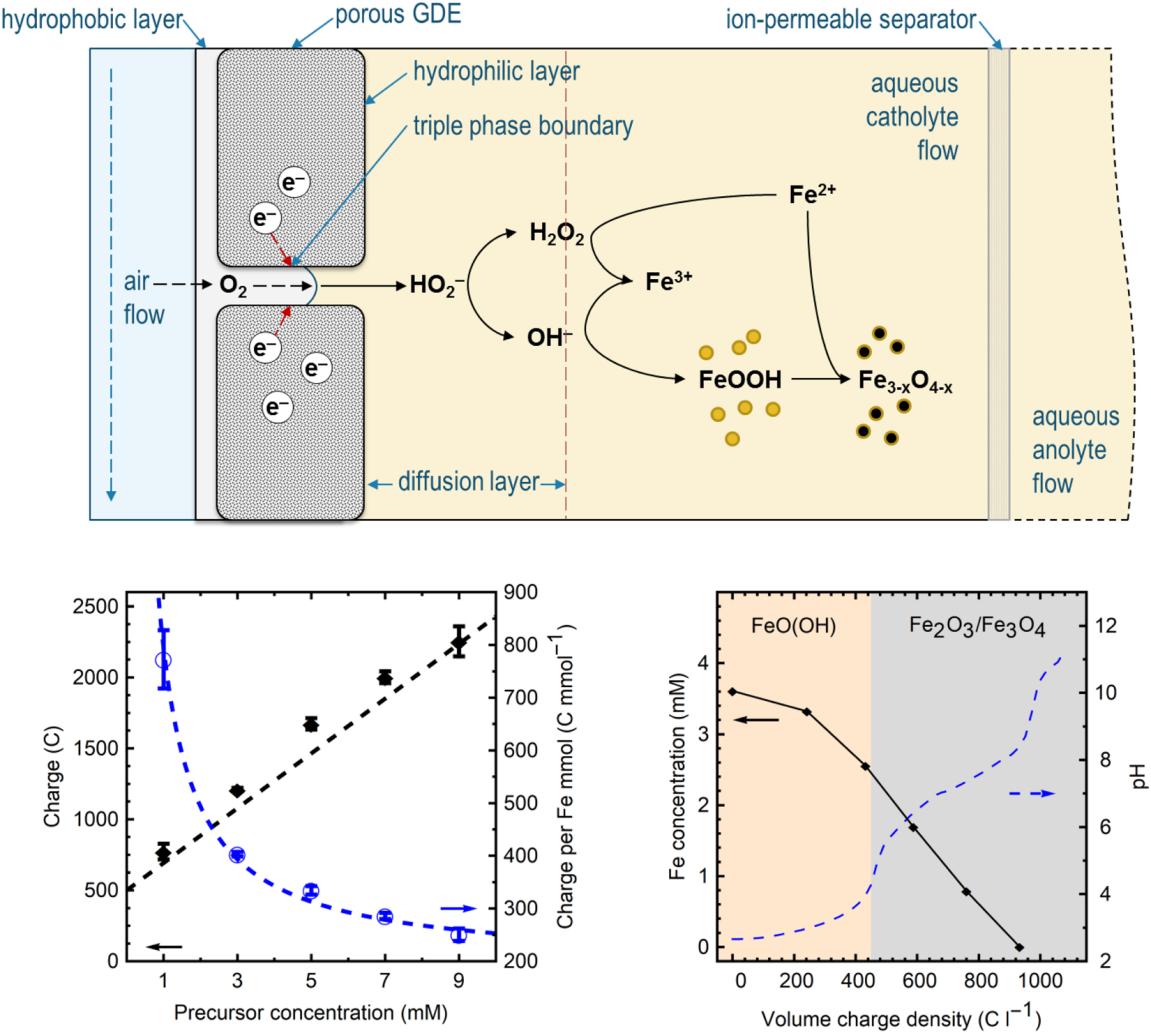
Top: Schematic of the triple interface at the gas diffusion electrode and the bulk solution showing the overall process and suggested mechanism of formation for the IONPs. Bottom left: Total charge applied (left) and charge per mol of Fe^2+^ precursor (right) for 5 synthesis conditions. Equation 7, for the total charge, and charge per mmol were used to model the dashed lines, respectively. Bottom right: Fe concentration profile and pH evolution during the GDEx synthesis for a solution of 5 mM FeCl_2_, the shaded regions denote the main phase formed at the different points of the process.

A GDEx experiment performed for the synthesis of these particles includes the elements shown in Fig. 1, at the cathodic interface. The electrochemical reactor itself contains 3 chambers. Through the first chamber, gas (e.g., air) flows at a fixed rate, with a set overpressure, at the hydrophobic layer of the gas diffusion electrode (GDE). The hydrophilic layer of the VITO CoRE^®^ (cold-rolled) GDE gives way to the catholyte chamber. The catholyte and anolyte flow from, and to, 3-necked glass bottles serving as reservoirs, through the respective cell compartment. The anolyte and catholyte in the cell are separated by a Zirfon^®^ ion-permeable separator. The anode is a platinum-coated disk. Both electrodes and the separator have a projected cross section of 10 cm^2^. The circuit is completed with a potentiostat, and a Ag/AgCl reference electrode is placed via a Luggin capillary close to the GDE.

#### Synthesis

IONPs were synthesized using concentrations of FeCl_2_ ranging from 1 mM to 9 mM. 0.25 mmol, 0.75 mmol, 1.25 mmol, 1.75 mmol and, 2.25 mmol of FeCl_2_·4H_2_O (99%, Sigma-Aldrich) were dissolved in deionized (DI) water, respectively, together with 7.5 g of NaCl to a total volume of 250 mL. The anolyte solution consisted of the same NaCl electrolyte without the Fe precursor salt. The starting pH of every solution was adjusted to 2.7 with 30 vol% HCl in water. The solutions were cycled through the GDEx cell with a peristatic pump (530, Watson-Marlow) at 42 mL min^−1^. Air was pumped through the gas compartment of the cell at 100 mL min^−1^, with an overpressure of 20 mbar(*g*). The solution and gas were flushed through the cell for 30 min prior to each experiment (without electrode polarization). A potential of −350 mV (vs. Ag/AgCl) was applied to the GDE using a Bio-Logic VMP3 potentiostat. At regular intervals, 1 mL samples of the catholyte solution were taken, centrifuged, and filtered with a 0.3 μm filter. The filtered solutions were analyzed with an inductive coupled plasma-mass spectrometer (ICP-MS) for the iron content. The pH of the catholyte was continuously monitored until a value of 11.5 was reached, at which point the polarization was stopped, and the suspension of particles was collected.

The suspension of particles as-synthesized was centrifuged 3 times at 12,000 rpm for 15 minutes, using a Sorvall RC 6+ centrifuge (Thermo Scientific). Each time, the particles were re-dispersed in DI water to clean any remaining NaCl and NaOH. This ensured near pure water resistivities (~18 MΩ) in the liquid phase. The samples were centrifuged one last time, and dried under a nitrogen atmosphere for further characterization.

### Characterization

#### X-ray diffraction (XRD)

The dry samples were analyzed by powder X-ray diffraction (XRD) in a PanAlytical X’Pert Pro diffractometer using a Cu Kα radiation source. Samples were crushed and placed in standard monocrystal sample holders. Measurements were performed with a spinner at 40 mA-40 kV spending 4 s per step with a step size of 0.04° 2θ in the 10–110° 2θ range. Rietvield refinements were performed in all samples to fit the profiles and extract the lattice parameters from the data using HighScore Plus software. Crystallite sizes were calculated using the Scherrer equation.

Additional XRD characterizations were performed in a PanAlytical Empyrean diffractometer using Co Kα radiation with 40 mA-45 kV, and a finer step size of 0.013° 2θ in the same scan range. The small step size and the Co source were chosen to probe the possibility of multi-phase detection by observing peak splitting at large angles.

#### Electron microscopy (SEM and TEM)

Micrographs of the dry samples were taken with a FEI Nova NanoSEM 450 instrument. Images presented were taken with secondary electrons and an acceleration voltage of 5.00 kV. The samples were mounted on a sticky carbon tab. With a Cressington HR208, a thin layer of about 2.5 nm of Pt/Pd (80–20 wt%) was sputtered on this stub, which was placed on the NanoSEM. A JEOL JEM 2200FS FEG transmission electron microscope was operated at 200 keV. The dry powder was dispersed in water and dropped on a copper TEM grid covered by a carbon film.

#### Dynamic light scattering (DLS)

A Zetasizer Nano ZS (Malvern) was used to perform Dynamic Light Scattering (DLS) measurements on colloidal suspensions. After synthesizing the samples, and removing excess salt and hydroxide by centrifugation as explained previously, but before drying, 1 mL aliquots of the produced dispersions were collected for DLS analysis. The concentrated samples were diluted in DI water to the range of 0.1 g mL^−1^ of particles to water. The pH was adjusted to 10 with a 1 mol L^−1^ solution of NaOH. A refractive index of 2.4 and an extinction coefficient of 0.13 were used for DLS measurements^17,18^.

#### Fourier transform infrared spectroscopy (FTIR)

The solid samples were measured in a Nexus^®^ Spectrometer (Thermo Nicolet). The powders were mounted on a stage with a diamond ATR for direct sampling of the materials.

#### Fe speciation

Permanganate titrations were performed to measure the concentration of ferrous ions in solution as the synthesis process occurs^19,20^. Samples from 1 mL to 5 mL were taken from the catholyte reservoir at various points during the synthesis. The samples were mixed with 1 mL concentrated HCl to dissolve any particles present and sonicated for 5 minutes under a nitrogen atmosphere. The mixture was diluted by adding 70 mL of distilled water. A 10 mM solution of NaMnO_4_ was prepared and used to titrate the iron samples.

#### Magnetic characterization

Hysteresis loops at 300 K, at a maximum field of 4000 KA m^−1^ were obtained by means of a VSM-PPMS 6000 Quantum Design magnetometer.

## Results and Discussion

A schematic of the electrode, triple interface, and bulk solution components used for GDEx are shown in Fig. 1, focusing on the cathode (half-cell) processes. The overall electrochemical reactions implicated are described below, the 4-electron (1) and 2-electron (2) oxygen reduction reactions in alkaline media^21^.

1 $${{\rm{O}}}_{2({\rm{g}})}+4{{\rm{e}}}^{-}+{{\rm{H}}}_{2}{{\rm{O}}}_{({\rm{l}})}\to 4{{\rm{OH}}}_{({\rm{aq}})}^{-}$$

2 $${{\rm{O}}}_{2({\rm{g}})}+2{{\rm{e}}}^{-}+2{{\rm{H}}}_{2}{{\rm{O}}}_{({\rm{l}})}\to 2{{\rm{OH}}}_{({\rm{aq}})}^{-}+{{\rm{HO}}}_{2({\rm{aq}})}^{-}$$

After polarizing the GDE at a constant potential, a steady state current density develops and the initially colorless solution progressively becomes light yellow; then, it proceeds to from a beige/brown dispersion. The concentration profile of iron in solution is shown together with the pH evolution (Fig. 1, bottom right) as a function of the applied electric charge. Approximately 7% of the iron is removed from the solution upon reaching a pH of 3. The color change can be attributed to the formation of ferric chloride complexes (reaction 4), followed by the early precipitation of iron(III) oxide hydroxide (reaction 5), FeOOH. This points to the oxidation of Fe^2+^ ions soon after the process starts (reaction 3), a result of the peroxide formed via the ORR (reaction 2).

3 $$2{{\rm{Fe}}}_{({\rm{aq}})}^{2+}+{{\rm{H}}}_{2}{{\rm{O}}}_{2({\rm{aq}})}\to 2{{\rm{Fe}}}_{({\rm{aq}})}^{3+}+2{{\rm{OH}}}_{({\rm{aq}})}^{-}$$

4 $${{\rm{Fe}}}_{({\rm{aq}})}^{3+}+3{{\rm{Cl}}}_{({\rm{aq}})}^{-}\to {{\rm{FeCl}}}_{3({\rm{aq}})}$$

5 $${{\rm{Fe}}}_{({\rm{aq}})}^{3+}+3{{\rm{OH}}}_{({\rm{aq}})}^{-}\to {{\rm{FeOOH}}}_{({\rm{s}})}+{{\rm{H}}}_{2}{{\rm{O}}}_{({\rm{l}})}$$

At a pH of 8.5, the Fe^+2/+3^ ions are fully removed from solution, and by the end point, the entirety of the iron has transformed into targeted precipitates. A common mechanism during co-precipitation processes involves the formation of goethite (Fe^III^OOH, reaction 5) followed by a topotactic transformation to magnetite, if in the presence of ferrous ions (Reaction 6)^22^.

6 $${{\rm{Fe}}}_{({\rm{aq}})}^{2+}+2{{\rm{FeOOH}}}_{({\rm{s}})}\to {{\rm{Fe}}}_{3}{{\rm{O}}}_{4({\rm{s}})}+2{{\rm{H}}}_{2}{{\rm{O}}}_{({\rm{l}})}$$

Regardless of the pathway, the total electric charge consumed to transform a mol of iron into a given precipitate composition is constant (constant slope shown in black trace of Fig. 1, bottom left). Such charge depends only on the pH change achieved, and the initial metal concentration. Current densities throughout the synthesis were constant for all precursor concentrations (80 A m^−2^), as well as for the blank electrolyte solution. Chronoamperometric data from experiments concerning each studied concentration can be found in SI Fig. 2. The volume charge density (*Q*_*t*_, C L^−1^) consumed by the synthesis can then be calculated by Eq. 7, as shown in Fig. 1 (bottom left, left axis). Furthermore, a more useful parameter—to determine when the total iron precursor has been transformed into a target precipitate—is that of the total charge, *Q*_*t*_, divided by the moles of iron present, *Q*_*t*_/*n*_*Fe*_ (C L^−1^ mmol^−1^) as shown in Fig. 1 (bottom left, right axis).

7 $${Q}_{t}={Q}_{0}/V+2{[F{e}^{2+}]}_{0}{\rm{F}}$$

where *Q*_0_ (C) is the charge required for the pH change from 2.7 to 11.5 (500 C L^−1^), [Fe^2+^]_0_ (mM) is the initial concentration of ferrous ions, *V* (L) is the total volume, and F is Faraday’s constant (96485 C mol^−1^).

The GDEx process is advantageous as it requires a single Fe^2+^ precursor, allowing a precise control of the reaction path, besides generating all necessary active reagents *in situ*, and consuming a low amount of charge, which translates into inexpensive processing. Very importantly, the use of precious metal catalysts is not needed to accomplish the targeted electrochemical conversions. Moreover, all the Fe^2+^ ion precursor supplied in the electrolyte can be fully exhausted from the solution, demonstrating a highly-efficient alternative.

### Electrosynthesis of ROS drives the oxidation of Fe^2+^

**Figure 2:**
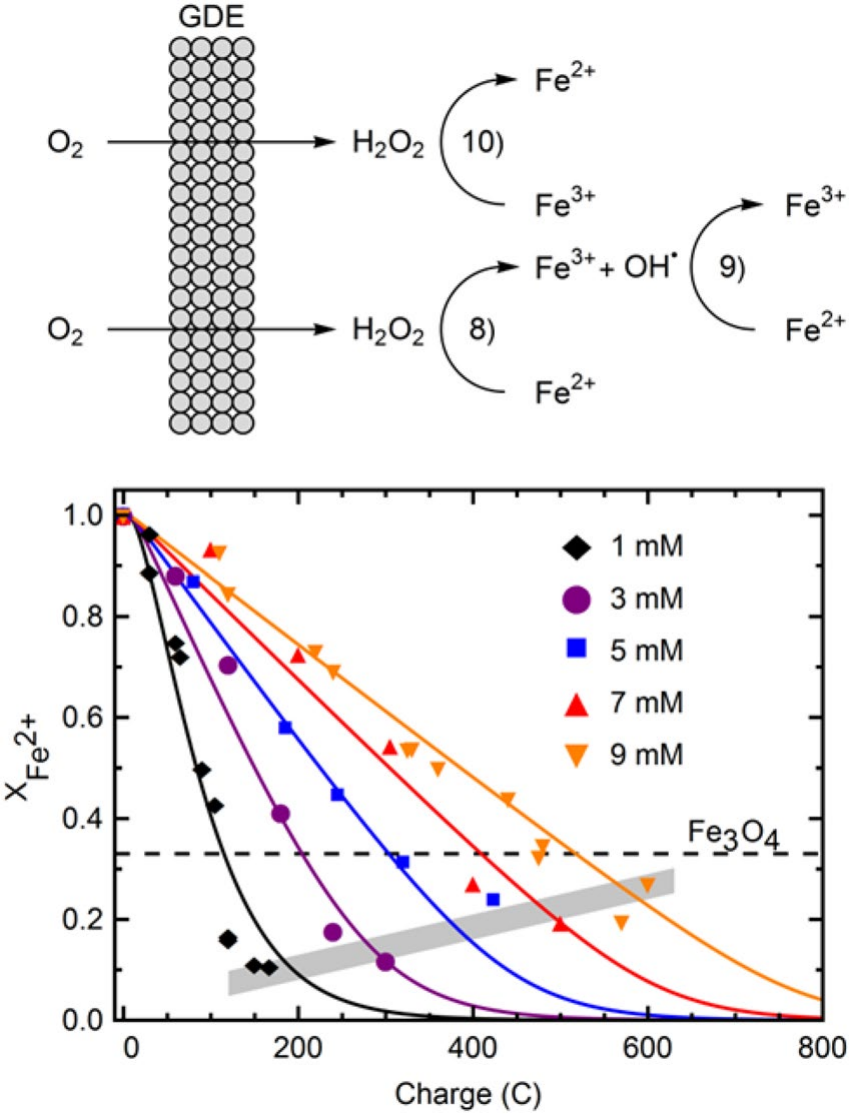
Top: Schematic of the modelled redox system for iron during the synthesis process, the relevant reactions are annotated. Bottom: Mole fraction of Fe^2+^ relative to total iron concentration vs the charge applied for 5 initial concentrations of iron precursor. The dashed line points at the ideal Fe(II) content of magnetite. The shaded region intercepts the curves at the points were samples were taken for further characterization.

The oxidation state of the iron ions in solution was studied with chemical redox titrations, by taking progressive samples throughout the synthesis process. The results of such chemical titrations for 5 key experimental conditions, i.e., increasing precursor concentrations, are shown in Fig. 2.

A systematic decrease in the concentration of Fe^2+^ from the pure precursor solutions was observed, until full depletion. Ultimately, all Fe^2+^ fully transformed into oxidized Fe^3+^ for all precursor concentrations studied. This is a significant step to achieve a precise control of the extent of iron oxidation and of the final redox features of the nanoparticles produced. It should be noted that no indications of Fe^2+^ transforming to Fe^0^ were detected, either in dispersion or at the surface of the GDE. The solid lines in Fig. 2 are modelled using an electro-Fenton approach for the reactions between peroxide and iron ions adapted from Qiu *et al*.^23^. By validating this model with our experimental data we demonstrate the feasibility to manipulate and control the oxidation of Fe^2+^ throughout the process, which translates into a precise control of stable oxidation states within the IONPs produced, which represents a challenge for materials engineering^24^. The main reactions considered for the model are shown below, and in the schematic of Fig. 2.

8 $${{\rm{Fe}}}_{({\rm{aq}})}^{2+}+{{\rm{H}}}_{2}{{\rm{O}}}_{2({\rm{aq}})}\to {{\rm{Fe}}}_{({\rm{aq}})}^{3+}+{{\rm{OH}}}_{({\rm{aq}})}^{-}+{{\rm{OH}}}_{({\rm{aq}})}^{\bullet }$$

9 $${{\rm{Fe}}}_{({\rm{aq}})}^{2+}+{{\rm{OH}}}_{({\rm{aq}})}^{\bullet }\to {{\rm{Fe}}}_{({\rm{aq}})}^{3+}+{{\rm{OH}}}_{({\rm{aq}})}^{-}$$

10 $${{\rm{Fe}}}_{({\rm{aq}})}^{3+}+{{\rm{H}}}_{2}{{\rm{O}}}_{2({\rm{aq}})}\to {{\rm{Fe}}}_{({\rm{aq}})}^{2+}+{{\rm{OH}}}_{2({\rm{aq}})}^{\bullet }+{{\rm{H}}}_{({\rm{aq}})}^{+}$$

11 $${{\rm{OH}}}_{({\rm{aq}})}^{\bullet }+{{\rm{H}}}_{2}{{\rm{O}}}_{2({\rm{aq}})}\to {{\rm{OH}}}_{2({\rm{aq}})}^{\bullet }+{{\rm{H}}}_{2}{{\rm{O}}}_{({\rm{aq}})}$$

The generation of peroxide at the electrode is taken from reaction 2, Eq. 7, and an efficiency factor (*η*) for the fraction of the charge that undergoes the 2-electron reduction process, over the 4-electron one. The model in Fig. 2 is shown with a current efficiency of 34%, while best fits of individual curves resulted in efficiencies between 30–36%. Thus, only a fraction of the total electric charge consumed is used to achieve the desired oxidation state of the Fe ions in solution. This is, only part of the O_2_ reduced at the electrode follows the 2-electron reduction to H_2_O_2_, whereas it is inferred that the rest follows the 4-electron path. Nonetheless, both paths contribute to achieving the targeted pH conditions at the different stages of the process; thus, a higher overall efficiency is implicit for the electrochemical transformations necessary to achieve the targeted products. The full model is provided in SI.

### Crystallite size control of single phase IONPs via precursor concentration

**Figure 3:**
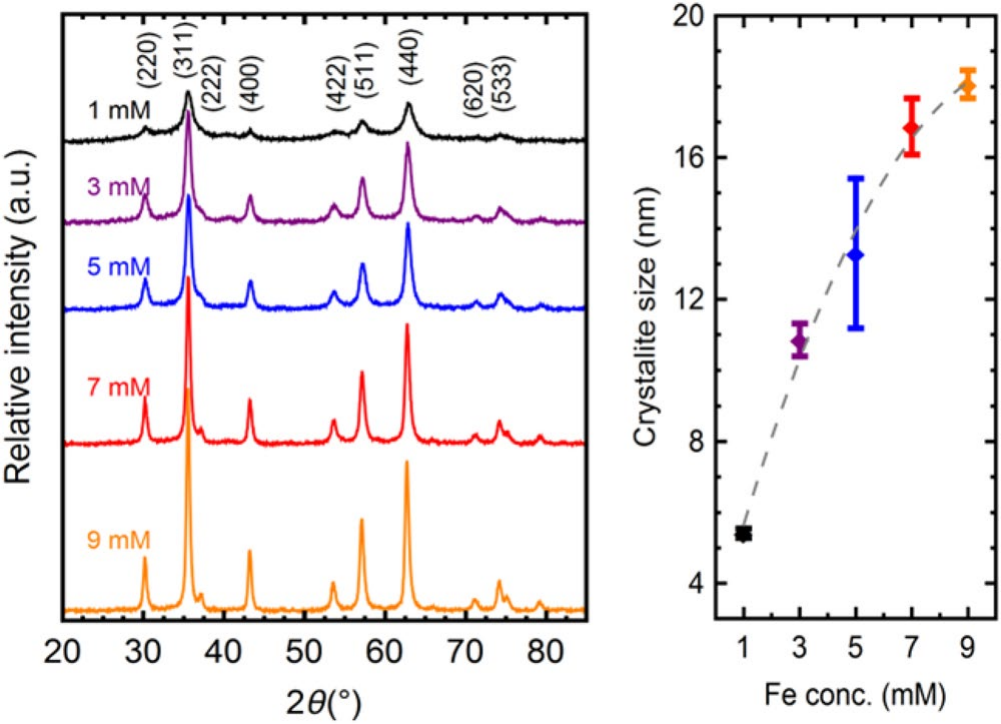
Left: X-Ray Diffraction patterns taken with Cu Ka radiation (λ = 1.540 Å) of samples synthesized from varying precursor concentrations of 1 mM, 3 mM, 5 mM, 7 mM and 9 mM, respectively. The peaks are indexed to the corresponding crystallographic planes of Fe_3_O_4_. Right: Crystallite size calculated from XRD data of the samples synthesized from solutions with FeCl_2_ concentrations 1 Mm through 9 mM. The dashed line is a guide to the eye.

With control over the charge needed to precipitate and carefully oxidize Fe(II) solutions to different degrees, 5 different synthesis conditions were further explored, with specific precursor concentrations of: 1 mM, 3 mM, 5 mM, 7 mM, and 9 mM. The diffractograms in Fig. 3 (left) show similar characteristics across all samples. The patterns are face centered cubic (fcc) inverse spinels of Fd3m space group, pointing to single phase, crystalline, Fe_3_O_4_. Nonetheless, total or partial lack of Fe^2+^ may be compensated by iron vacancies to form the structurally-similar maghemite (γ-Fe_2_O_3_)^4^. The differences between both oxides are not easily resolved by XRD and both phases are usually present in most magnetic iron oxide nanoparticles^25^. No peaks were found between 20° and 30° 2θ, and the (511) and (440) peaks showed no doublets; single peaks were found with both Cu and Co Kα radiation (SI Fig. 3) supporting the existence of a majority magnetite phase^25^.

FTIR spectra, shown in SI Fig. 4, reveal expected features for magnetite. All 5 samples exhibit the same absorption peak at 550 cm^−1^, indexed to vibrations from Fe-O bonds^26^. Samples synthesized from 3 mM Fe^2+^ solutions and under, especially from 1 mM, show O-H stretching vibrations (~3410 cm^−1^) and deformed vibrations (~1630 cm^−1^)^27^. The noise around 2200 cm^−1^ in all the traces arises from atmospheric CO_2_.

Several techniques were employed to determine the size of the particles and crystallites in different manners. The particles were transferred and analyzed in the dry state by SEM and XRD, while size distribution in dispersions was measured by DLS. Due to the nature of the techniques, crystallites, particles and aggregates can be studied to obtain a full characterization of the different sizes.

**Figure 4:**
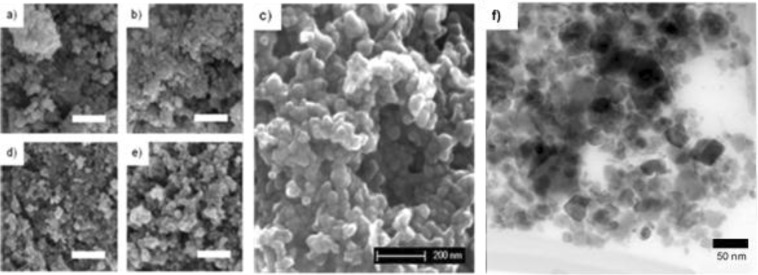
SEM Micrographs of samples synthesized from (**a**) 1 mM, (**b**) 3 mM, (**c**) 5 mM, (**d**) 7 mM and (**e**) 9 mM FeCl_2_ solutions. The white scale bars are 500 nm. (**f**) TEM image of a higher magnification of a 9 mM sample.

Significant peak broadening is observed in Fig. 3 (left), a feature of nanoscopic crystals^28^. The samples synthesized from a solution of 1 mM Fe^2+^ show the largest peak broadening, and a trend is clear: broader peaks appear for samples synthesized from lower iron precursor concentrations in GDEx. Crystallite sizes were calculated from the diffractograms using Scherrer equation^29^, the results are shown in Fig. 3 (right). The crystallite size was found to be controllable within a 5 nm to 20 nm range. At the opposite end of the spectrum, DLS measurements showed sizes 5 to 20 times larger than the individual crystallites (SI Figs 5 and 6), a common occurrence in bare IONPs as the hydrodynamic size of colloidal aggregates is measured^30^. Stable dispersions (|ζ-potentials|> 25 mV) were readily prepared and measured for the samples with lower coercivity (further explained), a feature of the transition from ferrimagnetic to superparamagnetic, as the minimized magnetization at rest diminishes aggregation. Mean sizes of approximately 120 nm were obtained with a PDI of 0.2. SEM was used to measure the particle size in the dry state. Figure 4 shows the micrographs of sample particles resulting from solutions of the 5 aforementioned concentrations of iron precursor, and a, higher resolution, TEM image. Individual particles of the same, roughly, hexagonal shape and similar size distribution with mean sizes in the range of 30–40 nm are seen. When used for biomedical applications, nanoparticles within this size range are likely to be eliminated with ease from biological systems^31^. For instance, cancer hyperthermia and drug delivery applications require magnetic oxide nanoparticles in the 20–50 nm range, with narrow size distributions^32^, as is the case for the particles produced here with GDEx.

### Charge-controlled stoichiometry of IONPs results in lattice parameter changes

**Figure 5:**
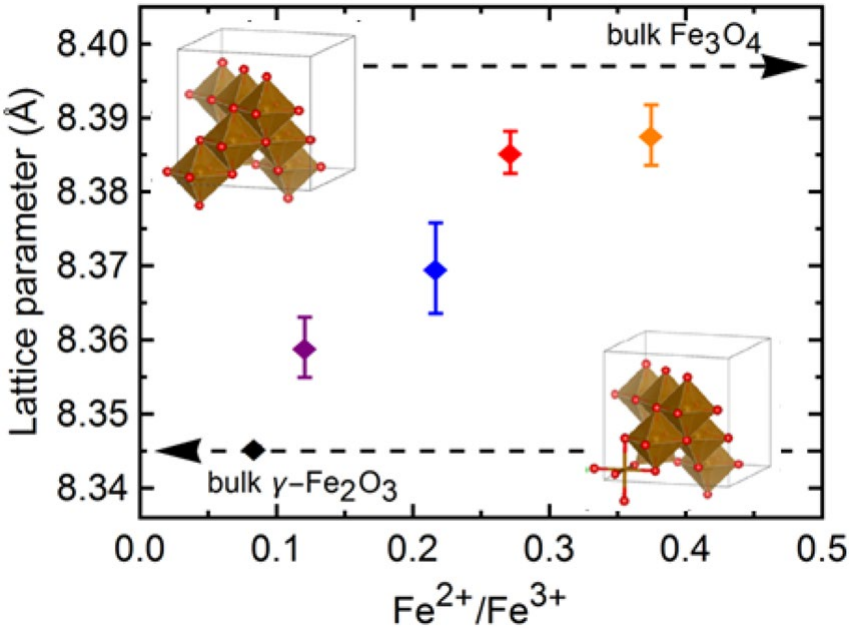
Lattice parameter vs iron content in the IONPs based on the Fe_3−x_O_4_ formula. The dashed lines are the reference values for bulk magnetite and maghemite respectively. The crystal structures reflecting each end state are drawn for visual reference using the 3D visualization software VESTA 3 (http://jp-minerals.org/vesta/en/)^43^.

An excess of oxidants may lead to a deficiency in Fe^2+^, and an over-oxidized material containing significant amounts of goethite or one of the ferric oxides. Control over the rate of production of oxidants is exerted with the applied charge. Rietvield refinements from the previously shown diffractograms yielded the lattice parameters shown in Fig. 5. A similar proportional trend as with the crystallite size is seen here. From 8.34 to 8.39 Å a large lattice expansion is seen when the samples are synthesized using a lower *Q*_*t*_/*n*_*Fe*_ (higher precursor concentrations). Changes in lattice parameters for metal oxide nanocrystals commonly arise from defects that distort the structure and introduce strain^33^. Reference values for the lattice parameters of γ-Fe_2_O_3_ (JCPDS 39–1346) and Fe_3_O_4_ (JCPDS 19–629) are presented with dotted lines in Fig. 5.

These values arise from an overall non-stoichiometry in the particles, deducing the presence of a γ-Fe_2_O_3_/Fe_3_O_4_ mixture^34^. The oxidation to γ-Fe_2_O_3_ from stoichiometrically co-precipitated Fe_3_O_4_ is common and expected^25^. Outermost layers of the magnetite NPs are commonly oxidized resulting in pseudo core-shell patterns or other mixtures^35^. The presence of more hydroxides groups, as seen in SI Fig. 4, can be attributed to the higher oxidation of the materials synthesized with a higher *Q*_*t*_/*n*_*Fe*_. An outer layer rich in ferric ions leads to hydration and the formation of oxyhydroxides^36^. Stoichiometric magnetite is composed of a face-centered cubic oxygen sub-lattice with Fe^3+^ ions in tetrahedral sites, and Fe^3+^ and Fe^2+^ in octahedral ones. Over-oxidation can be expressed as ferrous vacancies in the lattice. Exposure of Fe_3_O_4_ to oxygen can create such vacancies by surface oxidation and subsequent inward diffusion of defects. Structurally, magnetite can be written as (Fe^3+^)_tet_(Fe^2+^Fe^3+^)_oct_O_4_ with subscripts ‘tet’ and ‘oct’ referring to tetrahedral and octahedral sites respectively^37^. The oxidation of magnetite and the description of its defect structure is commonly described using O_2_ as the oxidant species^38,39^. The generation of vacancies (O_o_) and electron holes via other oxygen reactive species such as peroxide can be written in analogous way to that of diatomic oxygen (reactions 12 and 13). The outer overoxidized shell creates a lattice mismatch with the (nonstoichiometric-)magnetite core, inducing strain. This is compounded by the size mismatch of the vacant vs the occupied sites, as well as the ionic radii difference of the variable oxidation states of Fe.

12 $$2{{\rm{O}}}_{2}\to 4{{\rm{O}}}_{{\rm{O}}}+{{\rm{v}}}_{{\rm{tet}}}^{||}+2{{\rm{v}}}_{{\rm{oct}}}^{||}+8{\rm{h}}$$

13 $$2{{\rm{O}}}_{2}^{2-}\to 4{{\rm{O}}}_{{\rm{O}}}+{{\rm{v}}}_{{\rm{tet}}}^{||}+2{{\rm{v}}}_{{\rm{oct}}}^{||}+4{\rm{h}}$$

A thorough characterization of the stoichiometry or non-stoichiometry of IONPs is paramount, as it can lead to dramatic variations or even suppression of the Verwey transition temperature^32^, which associates to changes in the magnetic, electrical and thermal properties of the IONPs. Thus, as stoichiometric effects can be controlled via GDEx, the process ensures the possibility to determine and control precise structure-property relationships. This is exemplified in the following section, for the magnetic properties of the nanoparticles produced.

### Magnetization control

**Figure 6:**
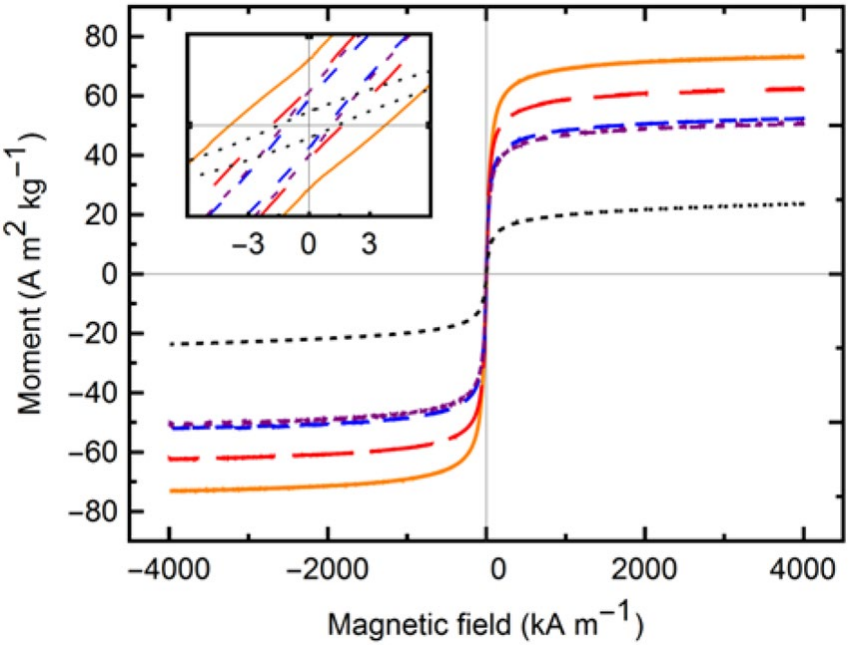
Magnetization hysteresis curves measured at 300 K in a VSM for samples synthesized with precursor concentrations 1 mM, 3 mM, 5 mM, 7 mM and 9 mM. The inset is a closer view of the origin to show the negligible coercivity obtained.

The magnetization curves are shown in Fig. 6 for 5 precursor concentrations. The curves show little hysteresis, the samples have small coercivity (Hc). The sample with the largest crystallite size (18 nm) exhibits also the largest coercivity, 3.9 kA m^−1^. The smallest coercivity (1.0 kA m^−1^) is seen with samples synthesized from 5 mM of Fe^2+^, corresponding to an 11 nm crystallite size. Samples with coercivity below 2 kA m^−1^ exhibited superparamagnetic behaviour when making dispersions. The maximum saturation magnetization (M_s_) is observed from the largest crystallites, ~80% that of bulk magnetite (taken as 92 A m^2^ kg^−1^). Table 1 displays the full results.

**Table 1:** Summary of structural and magnetic parameters.

Fe conc. (mM)	Crystallite size (nm)	Lattice parameter (Å)	*M*_*s*_ (A m^2^ kg^−1^)	*H*_*c*_ (kA m^−1^)
1	5	8.34	23.6	1.5
3	10	8.35	50.5	1.2
5	11	8.36	52.3	1.0
7	16	8.38	62.2	1.8
9	18	8.39	73.0	3.9

**Figure 7:**
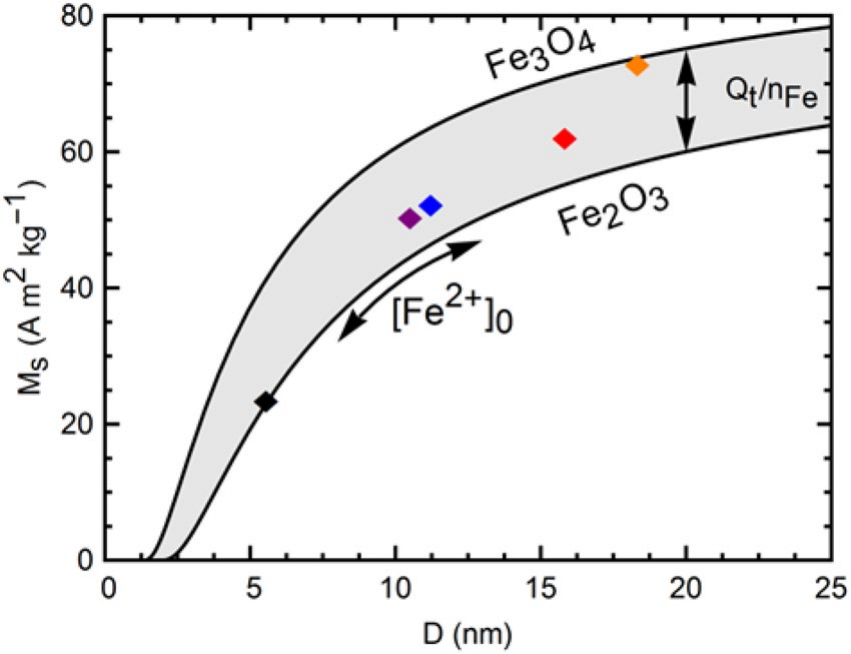
Saturation magnetization vs crystallite size in the IONPs for samples synthesized from precursor concentrations of 1 mM through 9 mM. The solid lines delimiting the shaded region are calculated curves from Eq. 14 for ideal magnetite and maghemite, as annotated. The arrows show the effect on the saturation magnetization of the initial precursor concentration, and the charge-to-mol ratio.

*M*_*s*_ is correlated to size; decreasing sizes commonly show decreasing magnetization. Spin canting, blocking layers, and surface oxidation may lead to decrease magnetization on the surface of particles. With smaller crystallite sizes, the surface composition becomes more significant to the bulk properties. Iron deficient magnetite, Fe_3−δ_O_4_, has a reduced bulk magnetization as well, down to 81 A m^2^ kg^−1^ for γ-Fe_2_O_3_. The samples synthesized with a higher *Q*_*t*_/*n*_*Fe*_ have a lattice parameter approaching that of bulk maghemite, and diminished *M*_*s*_. The combination of size and composition variations gives rise to the large range of saturation magnetization values observed, as shown in Fig. 7. Equation 14 is used to model the size-dependent saturation magnetization^40^, with the upper line and lower lines representing ideal magnetite and maghemite respectively.

14 $${M}_{s}={M}_{s,bulk}{(1-2d/D)}^{3}$$

where ${M}_{s,bulk}$ refers to the bulk saturation magnetization of either magnetite or maghemite, *D* is the diameter of the magnetic domain, an*d d* is the thickness of the non-magnetic disordered outer layer. Thus, a change in initial precursor concentration (leading to the reflected change in crystallite size) can move the obtained *M*_*s*_ along the calculated compositional lines, while changes in the charge-to-mol ratio (*Q*_*t*_/*n*_*Fe*_) determine the average Fe valence and as such the stoichiometry, moving the resulting saturation magnetization up or down in Fig. 7. As such, a synthesis map has been created to target the desired magnetic properties of the IONPs, a consequence of the structural and size parameters, which in turn are a result of the Fe^2+^ concentration (*n*_*Fe*_) in the feed solution and the charge applied (*Q*_*t*_) by GDEx.

The precise control of *M*_*s*_ is paramount for diverse applications, e.g., for high speed magnetization reversal in magnetic films and elements for use in magnetic storage and memory^41^, or for determining the spin-spin relaxation time *T*_2_ for use as contrast agents in functional magnetic resonance imaging^42^, among others.

## Conclusions

In conclusion, we have established an efficient and sustainable IONP synthesis method using a gas diffusion electrode. The novel process at hand, GDEx, shows remarkable control over the size, composition, and magnetization properties during the synthesis of IONPs. Using a single iron precursor, the oxidative nature of the GDEx process allows for easy control over these properties. A clear trend is seen between the charge applied and the resulting lattice parameters, strain-heavy IONPs are produced at higher charge/Fe experiments with compressed lattices further from the ideal value of bulk Fe_3_O_4_. Insight into the synthesis is obtained, from the removal of iron ions from solution, onset of precipitation, to the oxidative process of ferrous ions. Correlations between operational parameters such as initial concentration of Fe^2+^, and charge applied, were established with resulting material properties. GDEx represents a superior method for IONPs synthesis, and nanoparticle synthesis in general, as it is carried out at room temperature, with only sodium chloride as additive, and it uses the purest reagent: the electron. Ferrous chloride and air are passed through the electrode, representing a simple alternative with high possibilities for control and scale-up. GDEx presents an attractive alternative to reproducibly synthesize nanoparticles with minimal chemicals, and resources, especially with a view on biomedical applications due to the biocompatibility of its synthesis conditions.

## Supplementary information

Supplementary Figure
