# Supplementary material for: Gas Diffusion Electrodes on the Electrosynthesis of Controllable Iron Oxide Nanoparticles
Source: Sci Rep. 2019 Oct 25;9:15370. doi: 10.1038/s41598-019-51185-x (PMC6814830; doi:10.1038/s41598-019-51185-x)
Supplement: Supplementary file 1 — Supplementary Figure [file 41598_2019_51185_MOESM1_ESM.docx]

**Gas Diffusion Electrodes on the Electrosynthesis of Controllable Iron Oxide Nanoparticles**

Rafael A. Prato^ab^ Vincent Van Vught^a^ Sam Eggermont^bc^ Guillermo Pozo^ac^ Pilar Marin^d^ Jan Fransaer^b^ and Xochitl Dominguez-Benetton^ac*^

^a^ Sustainable Chemistry, VITO, Flemish Institute for Technological Research, Boeretang 200, 2400, Mol, Belgium.

^b^ Department of Materials Engineering, Surface and Interface Engineered Materials, Katholieke Universiteit Leuven, Kasteelpark Arenberg 44 - box 2450, 3001 Leuven, Belgium.

^c^ Strategic Initiative Materials in Flanders, SIM vzw, Technologiepark 935,BE-9052 Zwijnaarde, Belgium

^d^ Instituto de Magnetismo Aplicado, UCM-ADIF-CSIC, Universidad Complutense de Madrid A6 22,500 Km, 28230 Las Rozas, Spain.

Supplementary Information


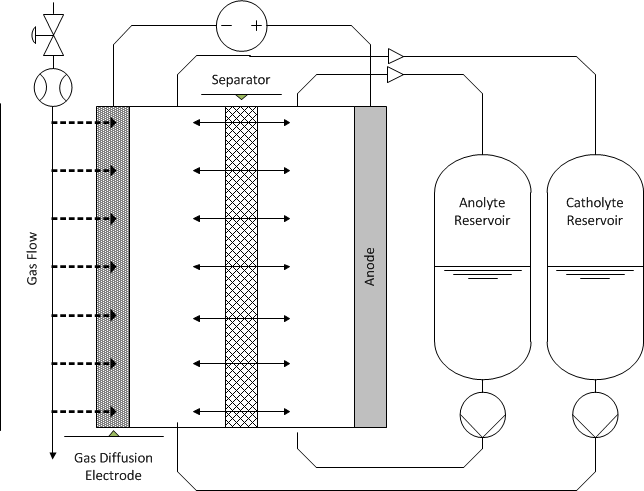


Figure 1. Schematic of the GDEx reactor used for the synthesis of all materials. The main components are annotated.


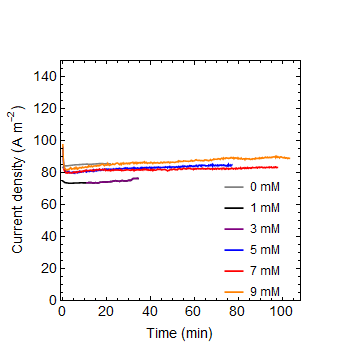


Figure 2. Chronoamperometric curves for experiments performed using only background electrolyte (0 mM) the 5 precursor concentrations studied.


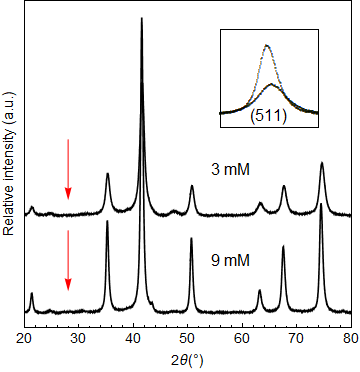


Figure 3. Difractograms of two samples synthesized by using 3 mM and 9 mM respectively under a Co-source XRD. The red arrows signal the position at which peaks are expected to appear for maghemite. The inset of the (511) peak shows a singlet.


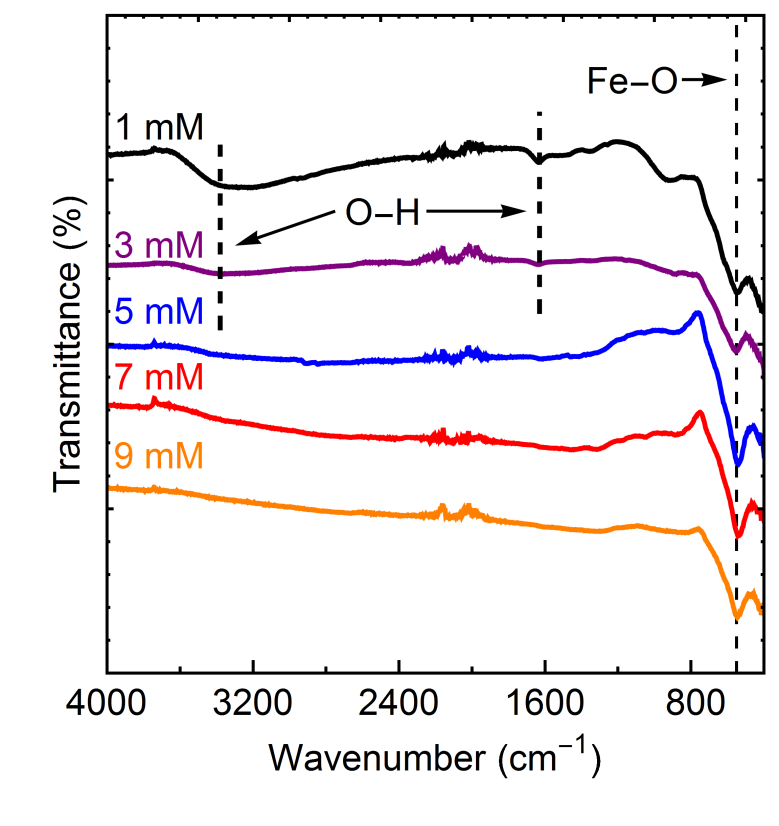


Figure 4. FTIR spectra from 5 samples synthesized at precursor concentrations of 1 mM, 3 mM, 5 mM, 7 mM and 9 mM. Highlighted are characteristic peaks indexed to Fe-O and O-H bonds.


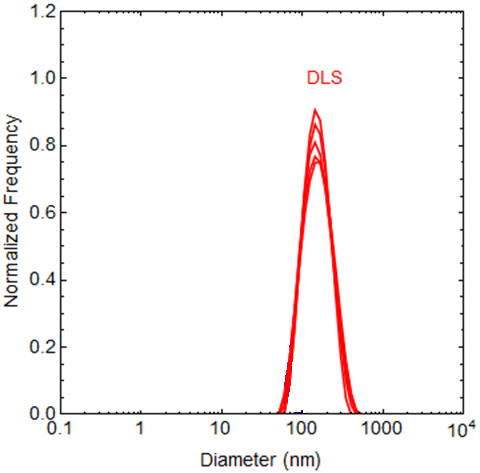


Figure 5. Sample DLS measurements for dispersion of the IONPs.


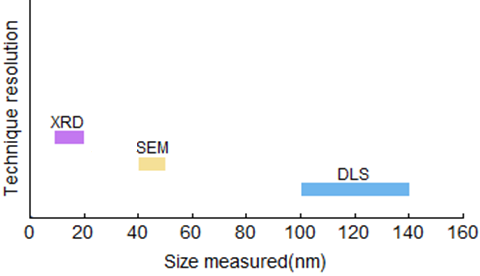


Figure 6. Comparison of the different techniques used for measuring the sizes of the cyrstallites (XRD), dry particles (SEM), agglomerates in water (DLS).

**Electro-oxidation model.** The model is adapted from reference 18. The subscripts *b* and *c* refer to the quantities in the bottle (reservoir) and cell (GDE electrochemical cell) respectively. τ is the delay time between the bottle and cell given by the the volumetric flow rate and the tube dimensions connecting the vessels. V is the volume of either vessel. Q is the volumetric flowrate. I is the current. F is Faraday’s constant. t is time. k are the rate constants for the respective reactions. η is the GDE coulombic efficiency towards producing hydrogen peroxide.

Table 1.

| $\mathrm{Fe}_{(aq)}^{2+}+H_{2}O_{2(aq)}\to\mathrm{Fe}_{(aq)}^{3+}+\mathrm{OH}_{(aq)}^{\boldsymbol{\cdot}}+\mathrm{OH}_{(aq)}^{\mathbf{-}}$ | 8 |
| --- | --- |
| $\mathrm{Fe}_{(aq)}^{2+}+\mathrm{OH}_{(aq)}^{\boldsymbol{\cdot}}\to\mathrm{Fe}_{(aq)}^{3+}+\mathrm{OH}_{(aq)}^{\mathbf{-}}$ | 9 |
| $\mathrm{Fe}_{(aq)}^{3+}+H_{2}O_{2(aq)}\to\mathrm{Fe}_{(aq)}^{2+}+\mathrm{HO}_{2(aq)}^{\boldsymbol{\cdot}}+H_{(aq)}^{\mathbf{+}}$ | 10 |
| $\mathrm{OH}_{(aq)}^{\boldsymbol{\cdot}}+H_{2}O_{2(aq)}\to\mathrm{HO}_{2(aq)}^{\boldsymbol{\cdot}}+H_{2}O_{(aq)}$ | 11 |

$$\frac{d{Fe}_{c}^{2+}}{dt}=\frac{Q}{V_{b}}{Fe}_{b}^{2+}\left( t-\tau\right)-\frac{Q}{V_{c}}{Fe}_{c}^{2+}\left( t \right)+{Fe}_{c}^{3+}\left( t \right)k_{10}H_{2}O_{2}\left( t \right)-2k_{8}{Fe}_{c}^{2+}\left( t \right)H_{2}O_{2}\left( t \right)$$

$$\frac{d{Fe}_{b}^{2+}}{dt}=\frac{Q}{V_{c}}{Fe}_{c}^{2+}\left( t-\tau\right)-\frac{Q}{V_{b}}{Fe}_{b}^{2+}\left( t \right)$$

$$\frac{d{Fe}_{c}^{3+}}{dt}=\frac{Q}{V_{b}}{Fe}_{b}^{3+}\left( t-\tau\right)-\frac{Q}{V_{c}}{Fe}_{c}^{3+}\left( t \right)-{Fe}_{c}^{3+}\left( t \right)k_{10}H_{2}O_{2}\left( t \right)+2k_{8}{Fe}_{c}^{2+}\left( t \right)H_{2}O_{2}\left( t \right)$$

$$\frac{d{Fe}_{b}^{3+}}{dt}=\frac{Q}{V_{c}}{Fe}_{c}^{2+}\left( t-\tau\right)-\frac{Q}{V_{b}}{Fe}_{b}^{3+}\left( t \right)$$

$$\frac{dH_{2}O_{2}}{dt}=\eta\frac{I}{F}-H_{2}O_{2}\left( t \right)({Fe}_{c}^{2+}\left( t \right)k_{8}+k_{10}{Fe}_{c}^{3+}\left( t \right)+\frac{k_{8}k_{11}H_{2}O_{2}\left( t \right)}{k_{9}})$$
